# Comparison between opportunistic and organised breast cancer mammography screening in the Swiss canton of Fribourg

**DOI:** 10.1186/s12885-019-5706-1

**Published:** 2019-05-17

**Authors:** Sarah Peisl, Stefan Zimmermann, Bertrand Camey, Daniel Betticher, Christine Bouchardy

**Affiliations:** 1 Department of Oncology, HFR Fribourg Hopital cantonal, CH-1708 Fribourg, Switzerland; 2 Cancer Registry of Fribourg, Rte St-Nicolas-de-Flüe 2, CH-1701 Fribourg, Switzerland; 3 Geneva Cancer Registry, IGH – University of Geneva, CH-1211 Geneva 4, Switzerland

**Keywords:** Breast cancer, Opportunistic screening, Organised screening, Mastectomy

## Abstract

**Background:**

Breast cancer screening mammography is widespread in industrialised countries within the framework of public health program or opportunist form. Only few data exist on the comparison of effectiveness between organised and opportunistic screening. The aim of this study is to compare organised and opportunistic screening using population-based data from the Fribourg cancer registry, Switzerland.

**Methods:**

We included all first primary breast adenocarcinoma diagnosed between 2006 and 2014 in women aged 50–69 years resident in the canton of Fribourg. We considered only breast cancer discovered by mammography screening. We compared patients, tumour characteristics and treatment modalities between breast cancer detected by the organised screening program versus opportunistic screening using logistic regression.

**Results:**

Out of 989 patients diagnosed with breast cancer, 402 (40.6%) were diagnosed by organised and 205 (20.7%) by opportunistic screening. Women with breast cancer detected within the screening program were more likely to be from rural areas (*P* = 0.035) and lived less frequently in high favoured regions (*P* = 0.020). They presented more frequently in situ than invasive cancer (*P* = 0.022). For patients with invasive breast cancer, those detected by the program were less likely to undergo mastectomy (*P* = 0.06) and consequently, they were more likely to undergo radiation therapy (*P* = 0.003). Adjustment for area of residence and financial context of the region did not modify the results presented.

**Conclusions:**

The present study reports an increased rate of detection of carcinoma in situ in organised screening program as compared to opportunistic screening mammographies, an indirect evidence of a higher radiologic sensitivity. Furthermore, the results show a trend towards more mastectomies among patients with breast cancer discovered after opportunistic than after organized mammography screening, reflecting lower treatment burden. Those results were independent of socio-economic factors which differed across screening groups.

## Background

Breast cancer (BC) is the most common cancer in Switzerland as well as in industrialised countries [1, 2]. Breast cancer mortality is decreasing in those countries due to the generalisation of mammography screening and improvements in adjuvant therapy [3]. Despite controversies regarding the balance between benefit and harms, especially overdiagnosis and overtreatment, the efficacy of breast cancer screening to reduce BC-specific mortality is well established [4, 5]. Mammography screening is now implemented in most European countries, either through public health programs and/or through opportunistic (individual) screening. Today, 12 Swiss cantons (Geneva, Vaud, Valais, Fribourg, Neuchâtel, Jura, Bern, Basel-Stadt, Thurgau, St-Gallen, Graubünden, Ticino) offer screening mammography as a public health service through an official program. These programs are coordinated at the national level by the Swiss cancer screening federation and are abiding to international guidelines of quality control [6, 7]. In Switzerland, as in other European countries, opportunistic and organised screening coexist with a large number of women in the same canton being screened outside the program due to the important offer of medical care in the private sector [8].

In the Swiss canton of Fribourg, a screening program was implemented in 2004, inviting all resident women between the age of 50–69 years (around 20′000 women) for screening every two years. The screening age has been extended to 74 years in 2015. The participation rate has stabilised around 55–63%, in line with the national average, and overall sensitivity of the screening program was estimated at 84% [9, 10]. A cancer registry established in 2006 provides data on breast cancers diagnosed within and outside the screening program.

While evaluation of screening efficacy for public health programs is extensively documented, there is only scare literature for opportunistic screening due to the of lack of robust centralised data of this individual approach [11–13]. The population-based cancer registry of Fribourg provides data which enables to distinguish between the mammography screening performed within the program from opportunistic screening and therefore evaluate the differences in efficacy between the two screening approaches.

The aim of this study, using population-based data from the Fribourg cancer registry, is to compare patients and tumour characteristics as well as treatment and overall survival between BC diagnosed through organised versus opportunistic mammography screening.

## Methods

### Data source and inclusion criteria

We included all first primary breast adenocarcinomas occurring among women aged 50–69 years registered in the Fribourg cancer registry, Switzerland between 2006 and 2014.

The cancer registry collects relevant information on patient’s tumour and treatment characteristics for all cancer occurring among the population resident in the canton. The data is acquired from various sources including hospitals, private practitioners, other cancer registries, pathology laboratories and more. It is then processed, verified for validity and completeness by and finally stored in an electronic database as coded data. All clinical records used for coding are attached to each case file. Case files are periodically updated, and active follow-up is performed routinely each year using the files of the Cantonal Population Office.

The registry regularly assesses survival. In brief, the index date refers to the date of confirmation of diagnosis or the date of hospitalisation if it preceded the diagnosis and was related to the disease. The exact cause of death is established from clinical records. Formal ethical approval and patient consent for this study was not required. The Fribourg cancer Registry has a general authorization to collect nominative data and to analyse the anonymized data.

### Patients

Socio-demographic variables such as country of birth, nationality, residential municipality and marital status are coded by the cancer registry according to the data available from the Cantonal Population Office. Municipalities are classified in urban and rural areas as a function of population density, number of inhabitants and accessibility according to the Federal Office for Statistics. The financial power of the municipalities is defined by the Cantonal Office for Municipalities as an index of 6 categories considering fiscal revenues, population density, workforce employment ratio and demographic growth. In this analysis, class 1 and 2 are summarized as categories of high, class 3 and 4 of middle and class 5 and 6 of low financial power.

Tumour stage is coded using the tumour, node and metastasis classification (TNM). Histological subtype and grade is coded using the International Classification of Diseases for Oncology (ICD-0-3). For this analysis, we classified the histological subtypes of invasive BC in ductal NST, lobular, ductal-lobular and other ductal carcinoma (e.g. apocrine, mucinous, papillary, tubular). Positive estrogen respectively progesterone-receptors ER/PR (defined as ≥10% of tumour cell staining), HER2/neu overexpression and the proliferation fraction MIB-1 are systematically collected.

The registry collects all treatments received within 6 months following diagnosis, in particular the type of surgery. The time to treatment is defined as the number of days between histological confirmation of breast cancer (invasive or in situ) and the start of the first treatment course.

The registry collects the presence of symptoms at diagnosis (yes, no, unknown), the mode of diagnosis (screening by mammography, clinical examination, self-examination and other diagnosis following symptoms, fortuitous discovery or diagnosis at death, unknown). We included only patients without symptoms at diagnosis and who had their cancer detected by mammography screening. After cross-checking the registry data with the database of the Swiss Cancer Screening Program, we divided patients in two groups, those who participated to organized screening program within 24 months before diagnosis i.e. “group organized screening” and those who did not i.e. “group opportunistic screening”. Interval cancers (i.e. occurring between two waves of mammography screening program) were all symptomatic and therefore excluded. The final cohort included 607 asymptomatic patients with screen detected breast cancer.

**Fig. 1:**
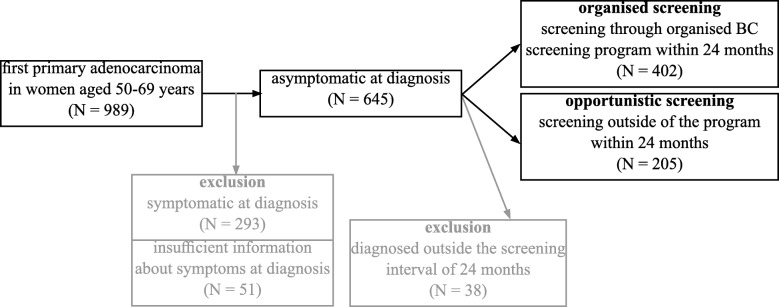
Breast cancer patient selection from the Fribourg cancer registry data 2006–2014

The selection of BC included in this study is summarised in Fig. 1.

### Statistical method

BC patients detected through opportunistic screening were compared to those detected through organised screening by logistic regression. We first considered the crude model. We secondly adjusted the model for all variables linked to patient characteristics showing significant differences, i.e. a *P-value* < 0.05, between the two screened groups in monovariate analysis. Those variables included the area of residence and the financial power of the area.

Overall survival of invasive BC was collated using Kaplan-Meier. The survival between the groups was compared by Log Rank test. No model selection was conducted. All statistical analyses were performed using IBM SPSS Statistics 23. A two-sided level of significance of 0.05 was used for all analyses.

## Results

Table 1 compares the demographic and socio-economic characteristics of patients diagnosed through opportunistic or organised screening. There was no significant difference in age, marital status, nationality, or country of birth between groups. Both groups shared similar socio-economic factors. Women from urban areas and areas of higher financial power, however, were significantly more likely to have been diagnosed through opportunistic screening than through organised screening (*P* < 0.035).

**Table 1:** Comparison of characteristics of breast cancer patients aged 50–69 years between organised and opportunistic mammography screening. Fribourg cancer registry 2006–2014

	Organised screening Number (%)		Opportunistic screening Number (%)		OR^a^	CI_95%_^a^	*P*-value
Age					1.0	[0.97; 1.02]	0.631
Mean (SD^a^)	59.62	(6.058)	59.92	(5.800)			
Marital status							
Married	246	(61.5)	128	(63.7)	1	[0.64; 1.29]	0.603
Other	154	(38.5)	73	(36.3)	0.9		
Nationality							
Swiss	366	(92.4)	183	(91.5)	1	[0.61; 2.11]	0.693
Other	30	(7.6)	17	(8.5)	1.1		
Country of birth							
Switzerland	320	(81.8)	154	(80.2)	1	[0.72; 1.72]	0.635
Other	71	(18.2)	38	(19.8)	1.1		
Linguistic area							
German	79	(19.7)	40	(19.5)	1	[0.66; 1.54]	0.967
French	323	(80.3)	165	(80.5)	1.0		
Area of residence							
Urban	225	(56.0)	133	(64.9)	1	[0.49; 0.97]	0.035
Rural	177	(44.0)	72	(35.1)	0.7		
Financial power of area							
High	84	(20.9)	64	(31.2)	1		0.020
Middle	190	(47.3)	86	(42.0)	0.6	[0.39; 0.90]	
Low	128	(31.8)	55	(26.8)	0.6	[0.36; 0.89]	

Calculated by logistic regression (reference: organised screening);

^a^ *OR* Odds ratio, *CI* Confidence interval, *SD* Standard deviation

Table 2 compares disease characteristics of cancers detected through opportunistic or organised screening. BCs did not differ significantly in tumour size, nodal involvement, presence of distant metastasis, histological and molecular subtype. However, BCs detected through the program were more likely to be diagnosed at the in situ stage (18.0% vs. 10.7%, *P* = 0.02). For invasive cancer the stage at diagnosis was similar between the program and the opportunistic screen.

**Table 2:** Comparison of characteristics of breast cancer among patients aged 50–69 years between organised and opportunistic mammography screening. Fribourg cancer registry 2006–2014

	Organised screening Number (%)		Opportunistic screening Number (%)		OR^a^	CI_95%_^a^	*P*-value
Type							
In situ	72	(18.0)	22	(10.7)	1	[1.09; 3.02]	0.022
Invasive	330	(82.1)	183	(89.3)	1.8		
Tumour size^b^							
≤ 20 mm	253	(77.8)	145	(80.6)	1	[0.54; 1.33]	0.476
> 20 mm	72	(22.2)	35	(19.4)	0.8		
T stage^b^							
T1mic-T1a	33	(10.2)	26	(14.4)	1	[0.36; 1.19]	0.518
T1b	85	(26.2)	45	(25.0)	0.7	[0.39; 1.25]	
T1c	135	(41.5)	74	(41.1)	0.7	[0.32; 1.19]	
T2-T3	72	(22.2)	35	(19.4)	0.6		
N stage^b^							
N0	252	(77.1)	134	(75.3)	1	[0.72; 1.69]	0.652
N+	75	(22.9)	44	(24.7)	1.1		
Metastasis^b^							
M0	326	(98.8)	176	(97.8)	1	[0.46; 7.50]	0.387
M1	4	(1.2)	4	(2.2)	1.9		
Histological subtype ^b^							
Ductal, NST	245	(74.2)	133	(72.7)	1	[0.32; 1.72]	0.543
Ductal, others	20	(6.1)	8	(4.4)	0.7	[0.60; 1.77]	
Lobular	43	(13.0)	24	(13.1)	1.0	[0.78; 2.91]	
Mixed	22	(6.7)	18	(9.8)	1.5		
Molecular subtype ^b,c^							
Luminal A	160	(53.5)	83	(53.5)	1		0.440
Luminal B, HER2-	75	(25.1)	36	(23.2)	0.9	[0.57; 1.49]	
Luminal B, HER2+	20	(6.7)	8	(5.2)	0.8	[0.33; 1.83]	
HER2+, non-luminal	11	(3.7)	12	(7.7)	2.1	[0.89; 8.97]	
Triple negative	33	(11.0)	16	(10.3)	0.9	[0.49; 1.80]	
ER/PR ^b,c^							
ER+/PR+	259	(78.5)	143	(78.5)	1	[0.40; 1.64]	0.693
ER+/PR-	27	(8.2)	12	(6.6)	0.8	[0.69; 1.93]	
ER−/PR-	44	(13.3)	28	(15.3)	1.2		
MIB-1 ^b,c^							
< 15%	172	(59.1)	92	(62.2)	1	[0.59; 1.32]	0.537
≥ 15%	119	(40.9)	56	(37.8)	0.9		
HER2^b^							
Positive	31	(9.5)	20	(11.0)	1	[0.47; 1.54]	0.595
Negative	295	(90.5)	162	(89.0)	0.9		

^a^Calculated by logistic regression (reference: organised screening);

^b^*OR* odds ratio, *CI* confidence interval;

^c^Invasive breast cancer only;

^d^*ER* estrogen receptor, *PR* progesteron receptor, *HER2* gene overexpression, *MIB-1* proliferation rate

Tables 3 and 4 compare treatment modalities of patients with carcinoma in situ and invasive breast cancer, respectively. There was no significant difference between women diagnosed with carcinoma in situ through opportunistic screening compared to women diagnosed through organised screening. For invasive BC however, patients diagnosed through opportunistic screening were more likely to undergo a mastectomy rather than breast conserving surgery in comparison with invasive breast cancer detected by organised screening (Odds ratio [OR]: 1.6, *P* = 0.06). However, this result was of borderline significance. As a result, women diagnosed through organised screening were significantly more likely to undergo radiation therapy (*P* = 0.003). There was no significant difference with regards to systemic therapy (chemotherapy, trastuzumab, anti-hormonal therapy). The time to treatment was similar for both groups.

**Table 3:** Comparison of in situ breast cancer treatment between organised and opportunistic mammography screening in women aged 50–69 years. Fribourg cancer registry 2006–2014

	Organised screening Number (%)		Opportunistic screening Number (%)		OR^a^	CI_95%_^a^	P-value
Breast Surgery							
Tumorectomy	57	(81.4)	18	(85.7)	1	[0.19; 2.86]	0.652
Mastectomy	13	(18.6)	3	(14.3)	0.7		
Radiation therapy							
Yes	48	(66.7)	15	(68.2)	1	[0.34; 2.60]	0.895
No	24	(33.3)	7	(31.8)	0.9		
Hormone therapy ^c^							
Yes	29	(40.3)	8	(36.4)	1	[0.44; 3.17]	0.742
No	43	(59.7)	14	(63.6)	1.2		
Time to treatment [days]							
Mean (SD^a^)	12.9	(9.3)	12.9	(7.6)			0.901^b^

Calculated by logistic regression (reference: organised screening);

^a^*OR* Odds ratio, *CI* Confidence interval, *SD* Standard deviation;

^b^*P*-value calculated by Mann-Whitney-U-Test;

^c^Treatment begun, but not necessarily completed

**Table 4:** Comparison of invasive breast cancer treatment between organised and opportunistic mammography screening in women aged 50–69 years. Fribourg cancer registry 2006–2014

	Organised screening Number (%)		Opportunistic screening Number (%)		OR^a^	CI_95%_^a^	*P*-value
Breast Surgery							
Tumorectomy	279	(85.1)	142	(78.5)	1	[0.98; 2.50]	0.060
Mastectomy	49	(14.9)	39	(21.5)	1.6		
Lymph-adenectomy							
Yes	85	(25.8)	45	(24.6)	1	[0.70; 1.62]	0.771
No	245	(74.2)	138	(75.4)	1.0		
Radiation therapy							
Yes	295	(89.4)	146	(79.8)	1	[1.29; 3.53]	0.003
No	35	(10.6)	37	(20.2)	2.1		
Hormone therapy ^c^							
Yes	278	(84.2)	147	(80.3)	1	[0.82; 2.09]	0.261
No	52	(15.8)	36	(19.7)	1.3		
Chemotherapy							
Yes	124	(37.6)	59	(32.2)	1	[0.86; 1.85]	0.227
No	206	(62.4)	124	(67.8)	1.3		
Trastuzumab^c^							
Yes	26	(7.9)	17	(9.3)	1	[0.44; 1.58]	0.581
No	304	(92.1)	166	(90.7)	0.8		
Time to treatment [days]							
Mean (SD^a^)	12.9	(8.0)	12.0	(8.2)			0.214^b^

Calculated by logistic regression (reference: organised screening);

^a^*OR* Odds ratio, *CI* Confidence interval, *SD* Standard deviation;

^b^*P*-value calculated by Mann-Whitney-U-Test;

^c^Treatment begun, but not necessarily completed

Additional adjustments for type of area and financial characteristics of regions did not modify the results.

**Fig. 2:**
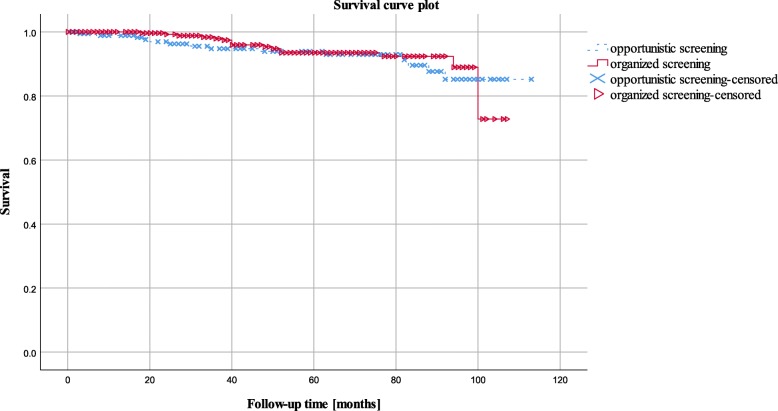
Overall observed survival of invasive breast cancer patients aged 50–69 years in organised and opportunistic mammography screening (Kaplan Meier Survival curve compared by Log Rank test). Fribourg cancer registry 2006–2014

Overall survival of invasive breast cancer patients did not differ significantly between patients diagnosed through organised and those diagnosed through opportunistic screening (94.8% vs. 92.3%, Hazard Ratio [HR]: 0.853, CI_95%:_ [0.42; 1.74], *P* = 0.656) (Fig. 2).

## Discussion

In this population-based study, organised and opportunistic screening were found to be equally effective with regards to the precocity of diagnosis of invasive BC and effect on overall survival. Nonetheless, this study finds a higher rate of in situ carcinomas in the organised screening group. Possible explanations for the significantly increased incidence of in situ carcinoma are higher sensitivity of mammography linked to the radiologists participating in the screening program who have specific training, high reading volumes, systematic access to previous mammographies, as well as systematic blinding of second readings. Neither the common use of concomitant ultrasonography in the opportunistic group, nor the higher screening intensity (commonly performed annually by private radiologists) were sufficient to compensate this difference.

In a previous study performed in Switzerland, opportunistic and organised screening provided little difference in prognostic profile in particular in terms of stage at diagnosis. However, BC prognostic factors were more favourable in Swiss regions covered by a program. In regions without a screening program, a higher prevalence of opportunistic screening was associated with better prognostic factors [11]. In contradiction to our study, the detection of in situ BC was similar between organised and opportunistic screening. In one rare study comparing breast cancers detected by organised and opportunistic screening in Loire-Atlantique, France, stages and biologic characteristics were largely identical between the two detection modes. Interestingly, one exception was a higher rate of in situ carcinomas in the opportunistic group, with a statistically significant OR of 1.29, in contradiction to our findings [13]. The authors of that study attributed this finding to higher frequency of screening in opportunistic screening. This hypothesis was not confirmed by our study.

The higher rates of in situ BC detection through organised screening could be considered both as a positive effect of screening or as a negative effect. The natural history of ductal in situ cancer is still ambiguous, and it is likely that some forms of DCIS would remain indolent during women’s life. While detection of DCIS is considered as a marker of screening effectiveness [6], it could also be considered as overdiagnosis which is one of the main argument raised in the current controversy regarding mammography screening [14, 15].

We found that socio-economic factors affected participation patterns. Women residing in urban areas and in communities with higher financial power were significantly more likely to be diagnosed through opportunistic than through organised screening. Previous studies have reported lower screening attendance among underprivileged women, indicators being education, income and poverty [16, 17]. A recent study performed in Switzerland comparing regions with and without organised screening programs showed no difference in socio-economic status between both groups. However, since opportunistic screening is widespread even in regions with an organised screening program, this study was unable to clearly differentiate between women participating in organised or opportunistic screening [18]. In accordance to our findings, a prospective study from Geneva showed that women undergoing opportunistic screening were facing a less challenging financial situation and had more contacts with a gynaecologist or general practitioner, suggesting better coverage of hard to reach population by organised screening programs [19].

Our study reports that treatment patterns of invasive cancers differed between diagnostic groups, with a borderline significantly higher rate of mastectomy in the opportunistic group. This imbalance is especially striking when the significant overlap of surgical teams is considered: cancer patients newly diagnosed through organised or opportunistic screening were largely managed by the same multidisciplinary teams for diagnostic workup, surgery, medical oncological therapy and radiotherapy. One possible explanation includes increased use of magnetic resonance imaging (MRI) in the opportunistic group, which could increase detection of multifocal lesions [20], and differences in tumour features not captured in the prognostic factors discussed above [21]. The negative correlation of screening participation and mastectomy rate has been previously described, but published studies were unable to distinguish organised from opportunistic screening [22].

Our study has limitation inherent to its observational nature. Even if we performed adjustment on several confounders, we could not exclude other bias such as difference in income or personal risk of breast (for example risk linked to positive family history of breast cancer) which is not recorded in our registry. Also we cannot identify women who previously participate to both opportunistic and organized program. Also length and lead time biases linked to screening are known to influence survival. However we believe that those biases occurred in both opportunistic and organized program and therefor do not impair survival comparison between the two groups.

Other main limitation is linked to the definition of opportunistic screening from the data cancer registry based on screening outside program among women with no symptoms at diagnosis. There is a possibility that women under surveillance for previous breast lesions or women with unreported symptoms were misclassified as having opportunistic screening. Given the fact that there is no centralised validated data on women with opportunistic screening, there was no alternative to better define opportunistic screening. Also, 5.2% of BC patients were excluded from the analysis due to a lack of documentation concerning symptoms at diagnosis, making group assignment impossible. In addition, as only diagnostic mammograms and organised screening mammograms, but not opportunistic screening mammograms, are covered by health insurances, the written indication for opportunistic screening could mention non-existent symptoms to ensure health care coverage, leading to an underestimation of the number of opportunistic screening exams. Finally, the power of observational studies in the Swiss cantons is low because of the small size of the inhabitants.

On the other hand, the study also has several strengths, beginning with the ability to separate diagnostic from opportunistic mammography based on individual medical files, and to accurately match population-based cancer registry data with program-based screening data. To our knowledge, this is the first study comparing treatments and socio-economic factors between women diagnosed through organised versus opportunistic screening.

## Conclusions

In conclusion, our study is one of the rare to provide information on effectiveness of opportunistic mammography screening in a population-based data set of an identical region (the Swiss canton of Fribourg). Both organised and opportunistic mammography provide similar precocity rates and mortality for invasive BC. However, we observed an increased rate of detection of in situ BC in organised screening mammographies as compared to opportunistic screening mammographies, an indirect evidence of a higher radiologic sensitivity. Furthermore, this study reports for the first time a reduced rate of mastectomy, which should be further confirmed.

## Abbreviations

BC: Breast cancer
ER: Estrogen receptor
ICD-0-3: International classification of diseases of oncology
NST: No special type
PR: Progesterone receptor
TNM: Tumour, node and metastasis classification
